# Development and psychometric study of the scale of the positive relationship PRIM + 19 in peruvian university students

**DOI:** 10.1186/s40359-023-01094-6

**Published:** 2023-03-02

**Authors:** Lindsey W. Vilca, Jannia M. Aquino-Hidalgo, Jhaleri Esteban-Brañes, Tomás Caycho-Rodríguez

**Affiliations:** 1 South American Center for Education and Research in Public Health, Universidad Norbert Wiener, Lima, Perú; 2 Departamento de Psicología, Universidad Peruana Unión, Lima, Perú; 3 Universidad Científica del Sur, Lima, Perú

**Keywords:** Positive relationships, Development of positive bonds, Management of interpersonal relationships, Integration, University students, Psychometric properties

## Abstract

**Background:**

Positive relationships are one of the most important components within the PERMA model since they facilitate the development of the other components. However, in the scientific literature, few instruments have been identified with solid psychometric properties that measure positive relationships in university students and adequately represent the construct. Therefore, the study aims to develop and study the psychometric properties of the PRI + 19 positive relationships scale through Confirmatory Factor Analysis, factorial invariance, and relationship-based validity with other variables.

**Method:**

A pilot sample of 201 university students (43.8% men and 56.2 women) between the ages of 18 and 34 (M = 20.9; SD = 2.74) was collected. The confirmatory sample consisted of 450 university students of both sexes (30.2% men and 69.8 women) between the ages of 18 and 35 years (M = 21.9; SD = 3.15). Along with the PRI + scale, other instruments were applied to measure satisfaction with life and psychological well-being.

**Results:**

In the pilot study, the Exploratory Factor Analysis showed the presence of three factors that could explain 54.5% of the items. In the confirmatory study, the Confirmatory Factor Analysis showed that the model of three dimensions related to 19 items presents the best adjustment indexes compared to other models (χ2 = 541.61; *df* = 149; CFI = 0.97; TLI = 0.97; RMSEA = 0.077 [IC90% 0.070 ‒ 0.084]). The scale also showed evidence of being strictly invariant for the groups of men and women. Finally, it was shown that the development of the positive bonds dimension positively predicts psychological well-being (0.35) and life satisfaction (0.20). The positive relationship management dimension positively predicts psychological well-being (0.28) and life satisfaction (0.29). Similarly, the integration dimension positively predicts psychological well-being (0.48) and life satisfaction (0.52).

**Conclusion:**

This study suggests that the PRIM + 19 scale is a useful tool from which valid and reliable interpretations of positive relationships in Peruvian university students can be obtained.

## Introduction

Positive relationships are one of the most important components within the PERMA model, as they facilitate the development of other components, such as positive emotions, commitment, purpose, and achievement [1]. In addition, this construct is positively linked with other Positive Psychology variables, such as life satisfaction, hope, gratitude, love, kindness, teamwork, resilience, and optimism [2–5]. Other studies have also shown that positive relationships are linked to high levels of well-being [6], with greater experience of positive emotions [7] and meaning of life [8]. On the other hand, positive relationships protect against negative factors such as stress, anxiety, loneliness, and depression [5, 9, 10].

However, this construct is not only linked to psychological and emotional aspects but also impacts people’s physical health. A meta-analysis carried out in 148 longitudinal studies showed that people with more positive and adequate relationships were 50% more likely to reduce the risk of mortality compared to those who had poorer or insufficient social relationships [11]. Another meta-analysis study showed that integration and social support significantly predict lower levels of inflammation in the body and, therefore, a better prognosis in the course of the disease [12].

Positive relationships can be defined as the ability to establish positive bonds, manage interpersonal relationships constructively and feel socially integrated, which includes feeling accompanied, cared for, supported, and satisfied with one’s social ties [4, 13]. Three important domains can be identified from this definition: (a) development of positive bonds, (b) management of interpersonal relationships, and (c) integration. The first domain refers to the ability of people to meet and establish positive bonds with new people. The second domain refers to the beneficial and constructive management of relationships with family and friends. This implies a close, kind, respectful behavior of mutual appreciation and joy for the triumphs of family and friends. These acts are important since they contribute to strengthening levels of self-esteem [14], mental health [15] and well-being [16]. The third domain refers to feelings of support, care, security, affection, and satisfaction that the person experiences with their family and friends. These experiences positively predict emotional well-being [17] and growth [18].

In this context, it is essential to adequately measure positive relationships, considering the three domains identified in its concept. Especially in the university population, since several studies have shown that not all students manage to establish positive bonds with their classmates and teachers [19–22]. Several studies show that isolation, loneliness, and lack of social integration are the main reasons for dropping out of university studies [23–25]. Concerning this, forming positive relationships between university students and their peers is crucial in establishing their identity in the university environment [26]. In addition, these social ties allow a successful adaptation to university life at a social and academic level [27–29]. In fact, these friendships are more profound, can replace family support, and reduce the likelihood of dropping out of college [30]. For all these reasons, it is important to evaluate the positive relationships in the university population adequately.

Concerning this, few instruments have been identified in the scientific literature that specifically measures positive relationships among university students. The first instrument was developed by Lacunza and Cotini [31], who proposed a brief five-item scale for adolescents, which identified two dimensions: (a) positive emotions and achievements linked to the practice of positive relationships; and (b) commitment to positive relationships. Although in the Exploratory Factor Analysis, both dimensions could explain 53.2% of the variability of the items, four items entered the first dimension, and the second dimension was only made up of one item, making the representativeness of this dimension impossible. In addition, the scale showed a low level of internal consistency (α = 0.51). Therefore, the scale only shows initial evidence of validity based on the internal structure and low reliability. No psychometric studies of the instrument in a university population were found.

Another study conducted on adults (18 to 85 years old) was found, where a brief scale of ten items was developed that measures two factors: (a) positive relationships and (b) achievement [32]. The model of two related factors in the study presented adequate adjustment indexes (χ^2^ = 82.1; *df* = 34; *p* < .001; CFI = 0.97; RMSEA = 0.06 [IC90% 0.04 ‒ 0.08]). Regarding its reliability, the test-retest correlation showed values above or close to 0.70 for up to 6 months. Although the scale shows adequate psychometric performance, the scale fails to separately identify and measure the three domains of the construct of positive relationships. In addition, most of the items on the scale measure disposition towards positive relationships and not the experience or practice of positive relationships (item 4: “What matters in life is being on good terms with other people”). On the other hand, no psychometric studies of the instrument on university students were found.

Positive relationships have also been evaluated as part of the PERMA profile; three items are raised in the test to measure positive relationships [33]. Although the scale has shown evidence of adequate psychometric performance in several studies, all of them have been carried out on adults between the ages of 18 and 90 [5, 34–37]. In addition, the PERMA profile fails to separately measure the three domains of positive relationships: development of positive bonds, management of interpersonal relationships, and integration. Also, in some studies, the items of positive relationships were included in another factor together with items of positive emotions and purpose [38, 39], evidencing that the items would not be able to differentiate the positive relationships construct of other factors of the PERMA profile. On the other hand, no psychometric studies of the instrument on university students were found.

For all the above, the present study has the following objectives: (a) develop a new scale to measure positive relationships, (b) evaluate the validity based on the content, (c) demonstrate the validity based on the internal structure, (d) evaluate the factorial invariance of the scale, (e) evidence the validity based on the relationship with other variables and (f) estimate the reliability of the scale.

## Method

### Participants

The pilot sample consisted of 201 Peruvian university students of both sexes (43.8% men and 56.2 women) between the ages of 18 and 34 (*M* = 20.9; *SD* = 2.74). The confirmatory sample consisted of 450 university students of both sexes (30.2% men and 69.8 women) between the ages of 18 and 35 years (*M* = 21.9; *SD* = 3.15). For the collection of both samples, a non-probabilistic convenience sampling was used using the following inclusion criteria: (a) informed consent of the participants, (b) being of legal age, and (c) studying for a university degree. The following exclusion criteria were also used: (a) not completing all the scales and (b) having a physical or sensory limitation that prevents answering the scales autonomously.

### Instruments

#### Positive relationships scale (PRIM + 19)

The scale has nigh teen items with four response categories ranging from (0) does not describe me to [3] describes me very well. In addition, the scale comprises three dimensions: Development of positive bonds, Management of interpersonal relationships, and Integration in the social circle. All the items are direct, where a higher score indicates a greater presence of positive relationships in their different areas.

#### Well-being index (WHO-Five)

For the study, the version adapted to Peru was used, aiming to assess the person’s subjective well-being [40]. The scale is made up of 5 items with 4 Likert-type response categories: (0) never, [1] sometimes, [2] often, and [3] always. In addition, there are no inverse items; therefore, a higher score on the scale represents a higher level of subjective well-being. Regarding its psychometric properties, in the adaptation study, the scale showed validity based on the internal structure (CFI = 0.99, RMSEA = 0.053, SRMR = 0.018) and good reliability values (ω = 0.76).

#### Life satisfaction scale (SWLS)

The scale was developed by Diener, Emmons, Larsen and Griffin [41]. The version adapted to Spanish was adopted for the study, which aims to evaluate the global judgment people make about their satisfaction with life [42]. This version shows evidence of adequate psychometric functioning in other studies conducted with Spanish-speaking university students [43, 44]. The scale is made up of five items with five response categories ranging from [1] totally disagree to [5] totally agree. The scale does not have inverse items; therefore, a higher score on the scale represents a higher level of satisfaction with life. Regarding the psychometric properties, the adaptation study showed that the one-dimensional model presented adequate adjustment indices (GFI = 0.98, NFI = 0.99, NNFI = 0.99) and an adequate level of internal consistency (α = 0.84).

### Development process of the PRI + 19 scale

**Fig. 1:**
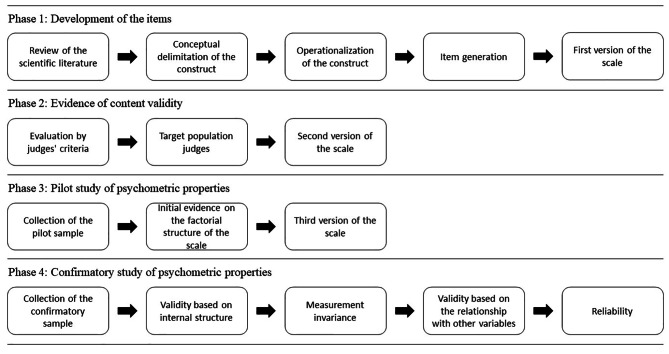
Development process of the PRI + 19 scale

The construction and evaluation of the psychometric properties were developed in four phases (see Fig. 1). In phase 1, the scientific literature was reviewed to define the construct conceptually. This process refers to the clear and precise definition of the construct, allowing the limits of the construct to be established and facilitating the development of the items [45]. For the conceptual delimitation, the definitions established in the PERMA model on positive relationships were used [4, 13, 46]. Then the operationalization of the construct was carried out through a qualitative approach. For which the Ventura-León [47] proposal was used to systematize the information, which contains four aspects: (a) Familiarization: all the definitions found are placed in a table to be studied; (b) Segmentation: the relevant information segments are identified; (c) Categorization: the information segments are ordered by similarity; (d) Correspondence: it is examined if the items are related to the categories generated previously. A deductive method was also used for elaborating the items, which implies an extensive review of the scientific literature and pre-existing scales [48]. This process identified three dimensions: the development of positive bonds, management of interpersonal relationships, and integration. The scale’s authors developed the items following the basic principles recommended in the scientific literature: representativeness, relevance, diversity, simplicity, and understandability [49]. The result of this first phase was the proposal of the first version of the scale.

In phase 2, content-based validity was assessed to ensure that the initial set of items adequately reflected the construct. For which the items were evaluated by four external judges, who evaluated the relevance, coherence, clarity, and unusual words in the Peruvian context. Afterward, the items were evaluated by 15 university students (target population), who evaluated the degree of clarity and left recommendations for a better understanding of the items. With the suggestion of both groups, modifications were made to the items, and a second version of the scale was proposed.

In phase 3, the initial study of the psychometric properties was carried out, for which a pilot sample large enough to draw valid conclusions about the factorial structure of the scale was collected. Several Exploratory Factorial Analyzes were carried out in this phase, where items were eliminated. The result of this third phase was the proposal of a third version of the scale. Finally, in phase 4, the scale’s psychometric properties were confirmed, for which a sample large enough to draw valid conclusions about the psychometric performance of the scale was collected. In this phase, evidence was shown on the Validity based on the internal structure, Measurement invariance, Validity based on the relationship with other variables, and reliability. The result of this last phase was the proposal of the PRI + 19 scale.

### Procedure

The standards are given in the Declaration of Helsinki were followed for the study [50]. Among this, the following principles were emphasized: (a) autonomy of the people to participate in the study, (b) respect towards the participants, (c) beneficence, and (d) justice to treat the participants with fairness and transparency. In addition, the study had the approval of the Ethics Committee of a private university in Lima (2021-CE-FCS - UPeU-00289). Informed consent was also used for the participation of people in the study.

In the pilot study, the sample was collected in the university campus environment. The average time to complete the surveys was 15 min. In the confirmatory study, the data was collected through a virtual form whose application was carried out in the virtual classrooms for approximately 15 min. The collection modality changed due to the Peruvian government’s restrictions on dealing with COVID-19. In both collection processes, the anonymity and confidentiality of the results were ensured, where the study’s objectives were explained to the university students, doubts related to the procedure were resolved, and they gave their informed consent to participate in the study.

### Data analysis

In the pilot study, Aiken’s V coefficient was used for content validity [51] and an ad hoc program in MS Excel© format was used for its computation [52]. Values greater than 0.70 were considered positive evaluations of the item [53]. For the initial study of the internal structure of the scale, the Exploratory Factor Analysis (EFA) was used using the method of Minimum Residuals (MinRes) with Oblimin rotation, and to determine the number of factors to extract, the Parallel Analysis was used [54]. For this, compliance with the basic conditions of the data was verified before performing the EFA. The Bartlett sphericity test and the Kaiser Meyer Olkin (KMO) index were used to verify the conditions.

In the confirmatory study, the Diagonally Weighted Least Squares with Mean and Variance corrected (WLSMV) estimator was used for the Confirmatory Factor Analysis (CFA) since the items are at the ordinal level [55]. The RMSEA, SRMR, CFI, and TLI indices were used to evaluate the fit of the models. For the RMSEA and SRMR indices, values less than 0.08 were considered acceptable [56]. For the CFI and TLI indices, values greater than 0.95 were considered adequate [57]. Cronbach’s alpha coefficient [58] and omega coefficient [59] were used to assess the reliability of the scale, where a value ω>0.80 is adequate [60].

The Multi-group Confirmatory Factor Analysis (MGCFA) was used to evaluate the factor invariance of the scale according to sex, where proposed a sequence of four hierarchical variance models: [1] configural invariance (reference model), [2] metric invariance (equality of factor loadings), [3] scalar invariance (equality of factor loading and intercept) and [4] strict invariance (equality of factor loadings, intercept and residuals). A formal statistical test was used to compare the sequence of models, for which the chi-square difference (Δχ2) was used, where non-significant values (p > .05) suggest invariance between groups. Second, a modeling strategy was used, for which the differences in the RMSEA (ΔRMSEA) were used, where differences less than < 0.015 show the invariance of the model between the groups [61].

An explanatory model was proposed regarding the validity of the PRI + 15 scale in relation to other variables. In this model, the dimensions of positive relationships significantly impact the level of satisfaction with life and psychological well-being. The WLSMV estimator was used to estimate the model, and the same adjustment indicators made in the Confirmatory Factor Analysis were taken into account.

The RStudio environment [62] for R [63] was used for the statistical analysis. Specifically, the “lavaan” package [64] was used to perform the CFA, the “semTools” package [65] to perform the factorial invariance, and the “mirt” package for the IRT models [66].

## Results

### Pilot study

#### Content-based validity

All the items presented good values in relevance (> 0.70), coherence (> 0.70), clarity (> 0.70), and context (> 0.70). However, following the recommendation of some judges, the content of 10 items was modified. After that, the clarity of the items was evaluated in a group of 15 students. Who reported that all the items were understandable and easy to understand. Therefore, there were no changes in the wording of the items.

#### Initial study of the internal structure

**Fig. 2:**
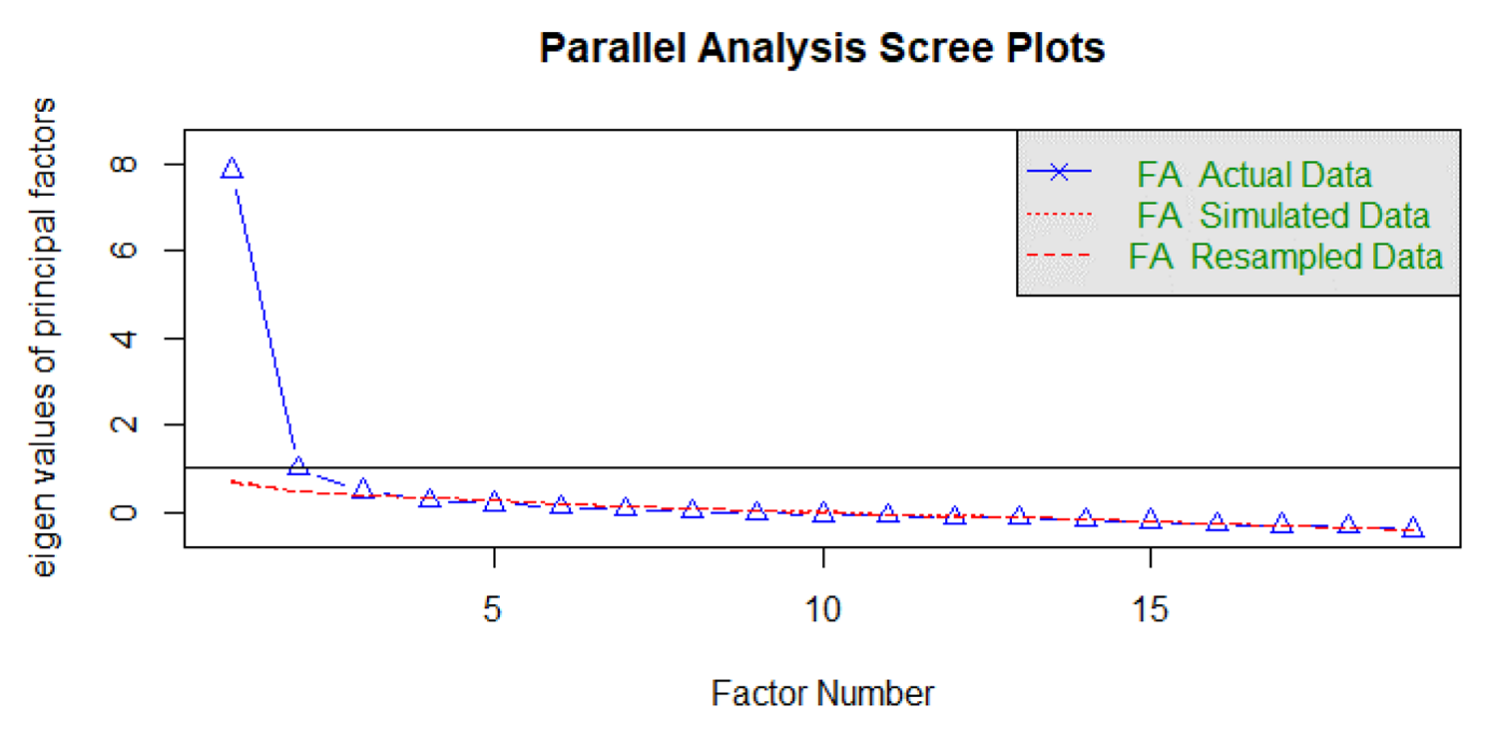
Parallel analysis of the set of items

In a first Exploratory Factor Analysis (EFA), items 4 (“I am happy with the relationship”), 14 (“I am tolerant with my friends and family, although I do not share their way of thinking and act”) and 20 (“I can establish a pleasant conversation with people I just met”) were eliminated since its content was very similar to other items on the scale. Also, they had a high factorial weight in a factor different from theirs. The decision was also made to join items 21 and 23 because their content was very similar. In a new exploratory factorial analysis, the parallel analysis estimated the presence of three factors (see Fig. 2) that were able to explain 54.5% of the variance of the set of items. This result coincided with the theoretical approach of the scale. Finally, there was a version of 19 items that were evaluated in the next stage.

### Confirmatory study

#### Descriptive analysis

Table 1 shows that item 14 (“I am happy for the triumphs of my friends and family.”) and item 10 (“I am respectful of the way my friends and family think”) present the average scores highest in the sample. That is, most of the participants agree with these statements. It can also be seen that item 16 (“I manage to establish a close bond with people I just met”) has the lowest average score. That is, most of the participants indicate that this statement describes them very little. It is also observed that all the items present asymmetry and kurtosis values within the expected limits (± 1.5).

**Table 1:** Descriptive analysis of the items

Items^a^	*M*	*SD*	*g*1	*g*2
1. I find it easy to make friends with people I have just met.	2.83	0.82	− 0.44	− 0.21
2. I am kind and attentive to people close to me (family and friends.	3.41	0.68	− 0.87	0.08
3. I feel that my friends and family spend time with me.	2.97	0.77	− 0.42	− 0.19
4. I enjoy spending time with my friends and family.	3.42	0.73	-1.13	− 0.72
5. I inspire confidence in people I have just met.	3.22	0.75	− 0.87	0.78
6. My relationship with friends and family is characterized by camaraderie and mutual appreciation.	3.37	0.68	− 0.87	0.62
7. I feel that my friends and family care about me.	3.26	0.74	− 0.86	0.65
8. When I am with my friends and family, I feel happy.	3.48	0.66	-1.07	0.76
9. When I meet new people, we quickly warm up.	2.72	0.82	− 0.24	− 0.44
10. I am respectful of the way my friends and family think.	3.52	0.63	-1.28	2.00
11. I can count on my friends and family in times of difficulty.	3.20	0.81	− 0.80	0.10
12. I feel satisfied with my relationship with my family and friends.	3.31	0.69	− 0.83	0.71
13. I am willing to help people I have just met.	3.27	0.73	− 0.67	− 0.17
14. I am happy for the triumphs of my friends and family.	3.64	0.57	-1.39	1.33
15. I feel accompanied by my friends and family.	3.22	0.74	− 0.70	0.25
16. I can establish a close bond with people I have just met.	2.70	0.82	− 0.17	− 0.49
17. My relationship with my friends and family helps me develop and achieve my goals.	3.23	0.74	− 0.70	0.09
18. I feel loved by my friends and family.	3.37	0.68	0.83	0.40
19. I get along well with people I have just met.	3.06	0.75	0.59	0.27

*Nota*. *M* = Mean; *SD* = Standard Deviation; *g1* = Skewness; *g2* = Kurtosis; ^a^ The translation of the items into English is only a translation made for study purposes

#### Validity base on internal structure

**Fig. 3:**
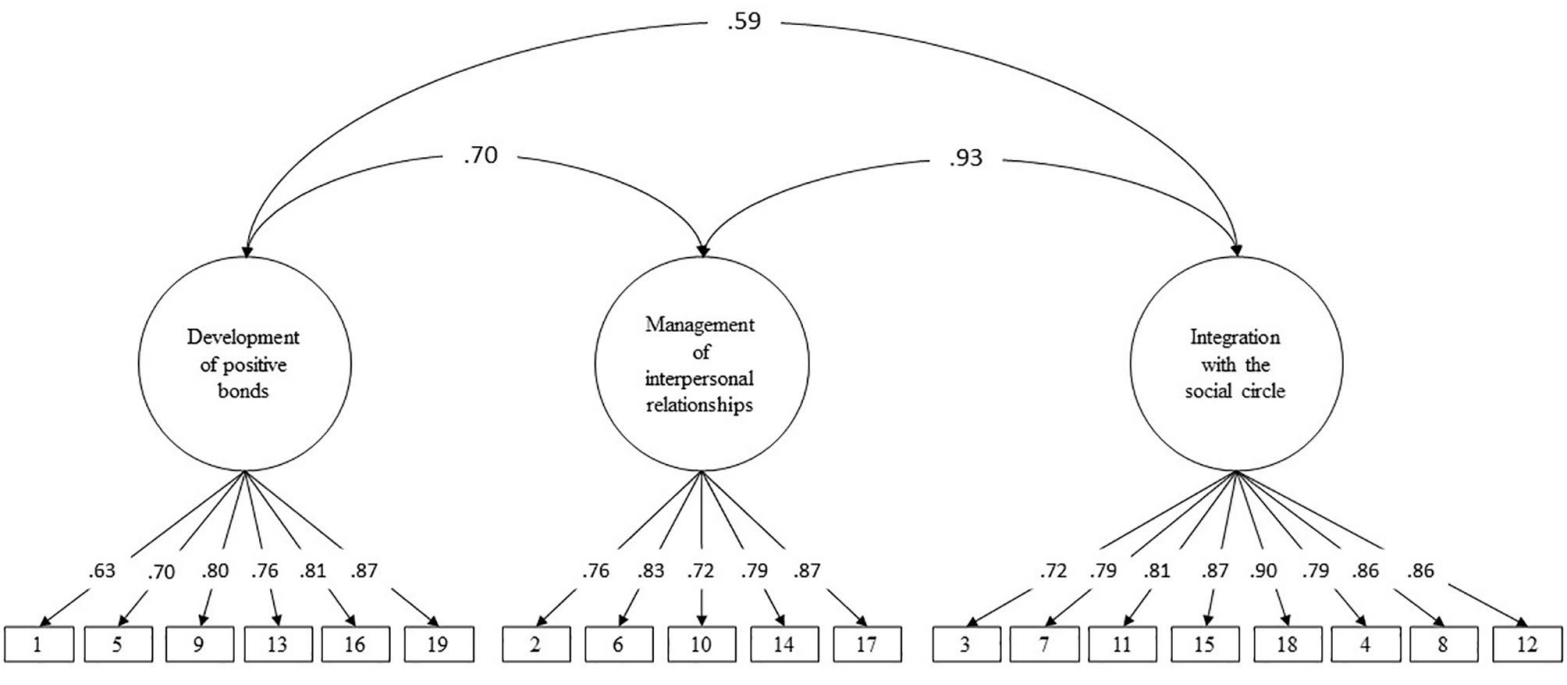
Confirmatory Factor Analysis of the PRI + 15 scale

The original model of three related factors (model 1) presented adequate fit indices (χ2 = 541.61; df = 149; CFI = 0.97; TLI = 0.97; RMSEA = 0.077 [IC90% 0.070 ‒ 0.084]). In addition, it can be seen in Fig. 3 that the factorial weight of the items was mostly high (λ > 0.70). However, it was found that the relationship between the Management of interpersonal relationships and Integration dimensions was very high (0.93). This result could suggest that both dimensions are very similar. Therefore, the fit of two competing models was evaluated. The model of two related factors (χ^2^ = 597.21; *df* = 151; CFI = 0.97; TLI = 0.96; RMSEA = 0.081 [IC90% 0.074 ‒ 0.088]) and the one-dimensional model (χ^2^ = 1533.59; *df* = 152; CFI = 0.90; TLI = 0.88; RMSEA = 0.142 [IC90% 0.136 ‒ 0.149]) presented lower adjustment indices than the original model. Therefore, the original model of three related factors was used for the following psychometric analyses.

### Factorial invariance by sex

Table 2 shows that the factorial structure of the scale has shown evidence of being strictly invariant for the groups of men and women in the sequence of proposed invariance models: metric invariance (ΔRMSEA = 0.005), scalar (ΔRMSEA = 0.000) and strict (ΔRMSEA = 0.000).

**Table 2:** *Adjustment indices of the PRI + 19 scale and invariance indices according to sex*

According to sex	χ^2^	df	*p*	SRMR	TLI	CFI	RMSEA [CI 90%]	Δχ^2^	Δdf	*p*	ΔRMSEA
Men	139.71	87	0.000	0.062	0.98	0.99	0.067 [0.046 ‒ 0.087]	‒	‒	‒	‒
Women	203.55	87	0.000	0.050	0.98	0.98	0.065 [0.054 ‒ 0.077]	‒	‒	‒	‒
Configural	231.46	174	0.002	0.044	0.96	0.96	0.038 [0.024 ‒ 0.051]	‒	‒	‒	‒
Metric	298.43	186	0.000	0.062	0.92	0.93	0.052 [0.041 ‒ 0.063]	29.26	12	0.004	0.014
Scalar	317.34	198	0.000	0.064	0.92	0.92	0.052 [0.041 ‒ 0.062]	21.09	12	0.049	0.000
Strict	347.12	213	0.000	0.072	0.92	0.92	0.053 [0.043 ‒ 0.063]	37.13	15	0.001	0.001

Nota: χ2 = Chi square; *df* = degrees of freedom; SRMR: Standardized Root Mean Square Residual; TLI = Tucker-Lewis Index; CFI = Comparative Fit Index; RMSEA = Root Mean Square Error of Approximation; Δχ2 = Differences in Chi square; Δdf = Differences in degrees of freedom; ΔRMSEA = Change in Root Mean Square Error of Approximation; ΔRMSEA = Change in Root Mean Square Error of Approximation

### Validity base on the relationship to other constructs

**Fig. 4:**
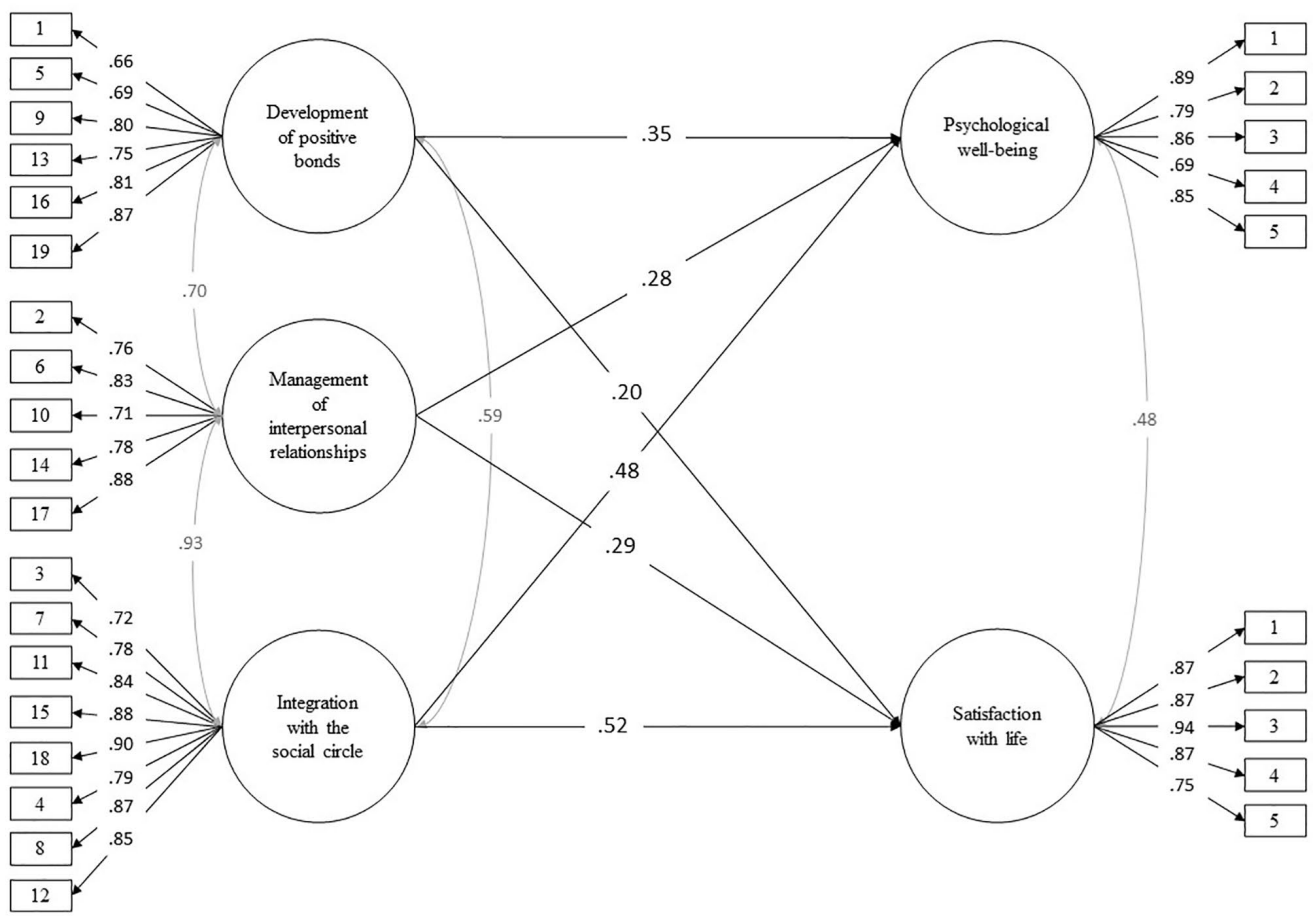
Predictive model of PRI + 15 on the level of psychological well-being and satisfaction with life

Considering the literature review, an SEM model was proposed to evaluate the impact of the dimensions of the PRI + 19 scale on the levels of psychological well-being and satisfaction with life. It was evidenced that the structural model presents adequate adjustment indices (χ2 = 867.1; *df* = 367; p = .000; RMSEA = 0.055[IC90% 0.050 ‒ 0.060]; CFI = 0.98; TLI = 0.98). In addition, the measurement models are adequately represented by their items since their factorial loadings are high in the factor that corresponds to them (see Fig. 4).

Figure 4 shows that the Development of the positive bonds factor has a significant and positive impact on the level of psychological well-being (0.35; p < .01) and the level of satisfaction with life (0.20; p < .01). It can also be seen that the management of interpersonal relationships factor has a significant and positive impact on the levels of psychological well-being (0.28; p < .01) and life satisfaction (0.29; p < .01). Finally, it is observed that the Integration factor has a significant and positive impact on the levels of psychological well-being (0.48; p < .01) and satisfaction with life (0.52; p < .01).

### Reliability of the scale

In the total sample of the study, the Development of positive bonds (α = 0.84[0.81 ‒ 0.87]; ω = 0.85[0.82 ‒ 0.88]), Management of interpersonal relationships (α = 0.83[0.80 ‒ 0.86]; ω = 0.84[0.81 ‒ 0.87]), and Integration (α = 0.90[0.89 ‒ 0.92]; ω = 0.92[0.91 ‒ 0.94]) dimensions present adequate reliability indices.

## Discussion

In the present study, the psychometric characteristics of the positive relationships scale PRIM + 19 in Peruvian university students were evaluated. Initially, the model of three related factors of 19 items was confirmed, which presented adequate adjustment indices. Therefore, it was shown that the Development of positive bonds, the management of interpersonal relationships, and integration in the social circle are adequate indicators of positive relationships in university students. These results coincide with the theoretical approach to the scale [4, 13]. he development of the positive bonds factor is important as it provides the basis for psychological well-being. However, when relationships are insecure or characterized by uncertainty, they can make it difficult for people to function properly [67]. Likewise, adequate management of interpersonal relationships also plays a fundamental role in the development and maintenance of mental health and well-being of people [15, 16]. Integration is another factor that positively predicts emotional well-being and personal growth [17, 18].

The current study also examined the measurement invariance of the PRIM + 19 by gender in college students. Thus, the scalar, metric and configurational invariance of the PRIM + 19 was maintained in the groups of men and women. These results indicate that the PRIM + 19 measures the same positive relationship construct for different sex groups. Therefore, it is likely that the pattern of sex differences in positive relationships is not explained by measurement bias but rather are real quantitative variations arising from psychological influences.

Likewise, the evidence of validity based on the relationship with other variables identified that positive relationships significantly predict psychological well-being and satisfaction with life. A greater establishment of positive relationships would predict greater well-being and satisfaction. This is expected since positive relationships are considered one of the pillars of well-being [68]. In this sense, it has been suggested that the chances of being happy increase by 15% if someone is related to another happy person [69]. Regarding the scale’s reliability, the study shows solid evidence of internal consistency in the total sample and the specific groups of men and women. This evidence guarantees a lower measurement error and greater score precision [70].

Among the study’s limitations, we can mention the use of self-report measures to obtain data. This type of measure evaluates the participants’ subjective perceptions and could exacerbate the common method’s variance due to a social desirability bias [71]. Therefore, it would be desirable to have a behavioral measure of positive relationships that can be used to investigate behavioral correlates of PRIM + 19 scores. Second, using non-probabilistic sampling would not allow the results to be generalized to the entire population of university students in Peru. The PRIM + 19 model could be tested using different regional samples of university students to generalize the results. Additionally, studies may use other cohort groups, which may improve the generalizability of the results. Third, the study’s cross-sectional nature does not allow the interpretation of psychometric results over time. In this sense, it is necessary to adopt longitudinal designs to assess to what extent the positive relationships construct could have comparable meanings over time. Fourth, other competing models, such as Bifactor or ESEM models, were not tested as the sample size used in the present study was insufficient to draw acceptable conclusions from these models. For the Bifactor model, sample sizes of 500 or more are recommended [72]. Regarding ESEM models, samples smaller than 500 increase the probability of inadmissible solutions [73]. Therefore, it is recommended for future research to study the factorial structure of the PRI + 19 scale under these models. Fifth, a Differential Analysis of the Items (DIF) was not carried out according to the sex of the participants. Therefore, it is suggested that future studies evaluate the invariance of the items from the IRT perspective.

Despite the limitations, the present study also has strengths. First, it provides new evidence for the concept of positive relationships in the university context of Peru, considering it in terms of the development of positive bonds, management of interpersonal relationships, and integration in the social circle. Second, the study shows a strength of a methodological nature since a detailed evaluation of its psychometric properties was carried out based on techniques derived from IRT and CTT. Third, PRIM + 19 responds to the need to have a measure of positive relationships that can be used locally and in nationwide surveys as part of programs that provide guidance services to university students. Fourth, the PRIM + 19 can be used in studies that help understand the background and results of positive relationships in the university context.

In conclusion, the present study suggests that the PRIM + 19 scale is a useful tool from which valid and reliable interpretations of positive relationships in Peruvian university students can be obtained.

## Data Availability

The datasets used and/or analyzed during the current study are available from the corresponding author on reasonable request.
